# The Role of Sex in Body Composition Differences in Hidradenitis Suppurativa: Insights from Bioelectrical Impedance Analysis

**DOI:** 10.3390/jcm14082760

**Published:** 2025-04-17

**Authors:** Zuzanna Piętowska-Marczak, Katarzyna Krefft-Trzciniecka, Alicja Pakiet, Danuta Nowicka

**Affiliations:** 1 Department of Aesthetic Dermatology and Regenerative Skin Medicine, Wrocław Medical University, 50-556 Wrocław, Poland; zuzia.pietowska@gmail.com (Z.P.-M.);; 2 University Center for General and Oncological Dermatology, Wrocław Medical University, 50-556 Wrocław, Poland; krefftkatarzyna@gmail.com; 3 Faculty of Physiotherapy, Wroclaw University of Health and Sport Sciences, 51-612 Wrocław, Poland

**Keywords:** hidradenitis suppurativa, body composition, inflammation, obesity, sarcopenia, muscle mass, fat mass, sex

## Abstract

**Background**: Hidradenitis suppurativa (HS) is a rare, debilitating, chronic inflammatory skin disease. This study aimed to investigate differences in body composition between patients with hidradenitis suppurativa (HS) and healthy controls, with a particular focus on sex-specific disparities, while also exploring secondary associations with muscle health and quality of life. **Methods**: Body composition was measured using a bioanalyzer and compared between HS individuals (*n* = 53) and controls (*n* = 50). **Results:** The mean BMI was significantly higher in the HS group than in the controls (median 29.6 vs. median 22.1; *p* < 0.001, effect size −0.581). The patients with HS had a significantly higher fat mass (mean 26.2 ± 22.7 vs. 16.3 ± 6.0; *p* < 0.001, effect size −0.400), level of visceral fat (median 9 vs. 2; *p* < 0.001, effect size −0.473), percentage of total body water (mean 45.9 ± 12.3 vs. 31.9 ± 14.3; *p* < 0.001, effect size −0.508), skeletal muscle index (median 8.9 vs. 7.3; *p* < 0.001, effect size −0.445), and bone mass (median 3.2 vs. 2.5; *p* < 0.001, effect size −0.421); at the same time, they had a significantly lower predicted muscle mass (median 19.8 vs. 47.3; *p* < 0.001, effect size −0.740) and percentage of skeletal muscle mass (mean 38.2 ± 7.8 vs. 42.3 ± 5.5; *p* = 0.008, effect size −0.263) in comparison to the controls. The HS group was also characterized by a higher metabolic age (median 65 vs. 21 years; *p* < 0.001, effect size −0.760) and basal metabolic rate (median 1927 vs. 1489 kcal; *p* < 0.001, effect size −0.444). **Conclusions**: Patients with HS exhibit a distinctive pattern in body composition parameters when compared to healthy controls, which may hold significant potential for enhancing diagnostic accuracy and monitoring disease progression. This study highlighted sex-specific differences in body composition, emphasizing the need to consider biological sex in the pathophysiology and clinical evaluation of HS. Further research is needed to explore the clinical utility of body composition analysis in disease progression, therapeutic response, and personalized management.

## 1. Introduction

Hidradenitis suppurativa (HS) is a chronic, inflammatory skin condition, classified as a neutrophilic dermatosis [1,2,3]. The disease most commonly begins in the early 20s, typically between the ages of 18 and 29, although it can occur in individuals of any age. Patients frequently report deep-seated, painful lesions that evolve into abscesses and fistulas, particularly in areas prone to friction and occlusion. These regions are susceptible to recurrent inflammation, which, over time, can lead to severe scarring and the formation of sinus tracts [4,5]. These lesions typically occur in areas rich in apocrine glands, such as the axillae, groins, and anogenital regions, where friction, moisture, and warmth exacerbate the condition. The etiology of the disease is multifactorial, primarily influenced by epigenetic factors that may affect keratinocyte differentiation. Additionally, hormonal imbalances, lifestyle and environmental factors, as well as immune dysregulation, all play a role in its complex pathogenesis [6,7,8,9,10,11]. Its pathogenesis remains incompletely elucidated, though it is believed to result from a complex interplay of genetic susceptibility, immune system dysregulation, and environmental influences [6].

HS is often associated with systemic comorbidities, including obesity, metabolic syndrome, and an elevated risk of cardiovascular disease, highlighting its impact beyond the skin [12,13,14]. Research [12] has highlighted a significant connection between HS and obesity, with body mass index (BMI) serving as a key factor in disease progression. Obesity contributes to meta-inflammation, a chronic inflammatory state that worsens HS by amplifying immune responses and skin lesions [15,16]. Studies [4,17,18] have consistently shown that obesity significantly exacerbates the severity of HS, leading to more frequent flare-ups, worse disease outcomes, and a marked decrease in patients’ quality of life. Individuals with HS describe its effects as debilitating, severely limiting their ability to perform daily tasks, maintain familial roles, and pursue professional activities. This profound burden underscores the need for holistic and individualized management strategies [12,19,20,21].

Cross-sectional studies revealed that patients with HS share similar risk factors to patients with chronic inflammatory diseases [1,15,19]. The literature extensively [4,12,17,22,23] documents the link between HS and obesity; however, a detailed assessment of skeletal muscle mass and adipose tissue distribution could offer valuable insights, as BMI alone provides a limited understanding of it and its role in HS pathophysiology. This limitation underscores the need for more detailed analyses of body composition beyond BMI to better understand how fat distribution and other parameters influence HS progression. In this study, we aimed to investigate differences in body composition between patients with HS and healthy controls, with a particular focus on sex-specific disparities. The rationale for focusing on those variables extends beyond its correlation with obesity. Fat allocation patterns such as visceral versus subcutaneous fat are known to differentially affect systemic inflammation and metabolic health [24]. Additionally, sex-specific hormonal variations may further modulate these effects; for instance, women with HS often exhibit higher fat mass and BMI compared to men, while men may show distinct alterations in muscle distribution [25,26,27]. These distinctions likely result from hormonal influences on inflammation and metabolism. Examining these disparities could help elucidate mechanisms underlying sex differences in HS prevalence and severity while informing targeted and more personalized interventions.

The prevalence and incidence of HS vary considerably across studies. HS affects approximately 0.00033% to 4.1% of the global population, with significant differences in prevalence across geographic regions [5,18,28,29,30,31,32]. Notably, the incidence of HS is higher in women, particularly among women of color. In Poland, the location of the present study, the estimated prevalence of HS is 1.6%, indicating a moderate occurrence within the country [33]. Gender medicine is an emerging field that examines how gender and sex influence disease patterns, pathophysiology, clinical presentations, and treatment outcomes. This area of research seeks to address sex-specific health disparities, aiming to improve diagnosis and therapeutic strategies by considering biological and social factors that affect men and women differently. Sex-specific differences are a notable feature of HS. In European and North American populations, HS disproportionately affects women, with a typical female-to-male ratio of approximately 3:1 [18,29,30,34,35]. In contrast, male predominance is seen in Asian populations, where the female-to-male ratio approaches 1:2 [36,37,38,39,40]. This discrepancy is believed to stem from a combination of genetic, hormonal, and environmental factors, including lifestyle differences such as smoking patterns between regions [27].

Additionally, women with HS often have a high BMI and fat mass, while men may exhibit distinct muscle distribution changes. These differences likely result from hormonal variations, such as sex hormones’ role in modulating inflammation and metabolism [12,25,41,42]. This is corroborated by Finnish research, which found that women of reproductive age are affected by HS at a rate twice as high as that of men, with the onset of new cases post-menopause being exceptionally rare [43]. The severity of the disease appears to fluctuate in correlation with the menstrual cycle. Studies have shown that exacerbations of HS are commonly observed during menstruation and the luteal phase, suggesting that hormonal fluctuations may influence the condition’s progression. This temporal relationship points to the potential role of estrogen, progesterone and androgens in modulating the inflammatory pathways involved in HS [41,42,44].

Despite growing awareness, HS remains underdiagnosed and challenging to manage. Further research is necessary to elucidate its pathophysiology, address sex-specific disparities, and explore novel therapeutic interventions. Therefore, the aim of our study was to investigate the differences in body composition between patients with HS and healthy controls, with a particular focus on sex-specific disparities. An additional aim was to explore muscle health and quality of life in those patients and their associations with body composition measurements. This approach sought to address existing gaps in the literature and enhance our understanding of how this chronic inflammatory condition differentially affects body composition in males and females.

## 2. Materials and Methods

### 2.1. Patients—Disease Severity and Quality of Life Questionnaires

This cross-sectional study included 103 participants: 53 HS patients (diagnosis confirmed by dermatologists via clinical criteria) and 50 controls without HS. Participants were recruited at the University Centre of General Dermatology and Oncodermatology in Wrocław, Poland.

The inclusion criteria for the study were as follows:

- Adults ≥ 18 years.
- Willingness to undergo bioelectrical impedance analysis (BIA) measurements.
- HS patients: Active Hurley Stage I–III lesions (dermatologist-confirmed HS diagnosis).
- Controls: No history of HS or other chronic inflammatory dermatoses.

The exclusion criteria were as follows:

- Pregnancy or lactation.
- Medical implants (pacemakers, monitoring devices).
- Severe mobility impairments.
- Concomitant treatments: Systemic antibiotics, biologic therapy, corticosteroids, or retinoids within 4 weeks prior to study.
- Comorbidities: Dialysis-dependent renal failure, decompensated heart failure, or electrolyte imbalances.
- Recent significant weight fluctuation (>5% body mass in prior month).

Disease severity assessment was performed using the Hurley and International Hidradenitis Suppurativa Severity Score System (IHS4) scales. The Hurley staging system classifies patients based on the presence and magnitude of clinical inflammation and exudation [7,45]. Hurley Stage 1 is characterized by solitary, isolated lesions, such as nodules or cysts occurring without the presence of sinus tracts or scarring. Stage 2 progresses to include recurrent lesions with the formation of sinus tracts and scarring. Stage 3 represents the most advanced and severe form, marked by extensive and confluent lesions, widespread sinus tract formation, and significant fibrosis and scarring across multiple regions. The International Hidradenitis Suppurativa Severity Score System (IHS4) quantifies disease severity by assigning weighted scores: each nodule counts as 1 point, each abscess as 2 points, and each draining tunnel as 4 points. The total score categorizes the severity of HS as follows: ≤3 indicates mild disease, 4–10 signifies moderate disease, and a score of 11 or higher reflects severe disease [46]. The control group was recruited through an institutional advertisement (poster) displayed within our institution and screened to exclude HS or chronic inflammatory conditions. As an incentive, all study participants were offered complimentary anthropometric and body composition measurements.

Prior to participation, all individuals provided written, informed consent. The study adhered to ethical guidelines outlined in the Declaration of Helsinki by the World Medical Association. Furthermore, the research protocol received approval from the Bioethics Committee at Wroclaw Medical University, Poland (approval no. KB-421/2022).

The assessment of quality of life was conducted using the DLQI (Dermatology Life Quality Index) and HiSQOL (Hidradenitis Suppurativa Quality of Life) questionnaires. These validated tools are commonly employed in dermatological research to evaluate the impact of skin diseases on patients’ daily functioning, emotional well-being, and overall quality of life. The Dermatology Life Quality Index (DLQI) is a 10-item questionnaire that evaluates patients’ perception of their skin disease’s impact on their health-related quality of life. The recall period is 7 days. The patients can score between 0 and 30 points, with 0–1 signifying no effect at all; 2–5 signifying a small effect; 6–10 signifying a moderate effect; 11–20 signifying a very large effect; and 21–30 signifying extremely large effect on patient’s life [47]. The Hidradenitis Suppurativa Quality of Life Questionnaire (HiSQOL) is a 17-item questionnaire used to assess the quality of life of HS patients, with a recall period of 7 days. The patients can score for 0–68 points with 0–4 signifying no impact; 5–14 signifying mild impacts; 15–21 signifying moderate impacts; 22–23 signifying severe impacts; and 24–64 signifying very severe impacts on quality of life [48]. Scores were analyzed in relation to body composition parameters.

The SARC-F questionnaire was used to assess the risk of sarcopenia in the study participants. This tool evaluates key aspects, such as strength, assistance with walking, rising from a chair, climbing stairs, and falls, to determine the likelihood of sarcopenia. The total number of points to obtain is 10, and ≥4 points indicates suspected sarcopenia and a need for further, more comprehensive evaluation [49].

### 2.2. Body Composition Measurement

BIA was performed using the TANITA MC-780MA bioanalyzer (Tanita, Tokyo, Japan) [50]. This device is a multi-frequency, mobile body composition analyzer, designed at a scale appropriate for professional use. The device uses three frequencies (5 kHz, 50 kHz, and 250 kHz) to assess body composition parameters, including fat mass, fat-free mass, total body water, and segmental analysis. The measurement process involves the use of four electrodes embedded in the platform, through which an electric current is passed. The current analyzes the body composition within 20 s, with the results either printed out or transmitted to a computer for further analysis. The manufacturer specifies that the accuracy of the measurement for body weight is within ±100 g.

### 2.3. Study Procedure

On the day of the examination, participants were instructed to have a light breakfast, with a minimum of four hours between eating and the examination, and one hour between drinking and the examination. They were also advised to refrain from physical activity (24 h prior to BIA) and smoking on the day of the test. All measurements were conducted between 1 PM and 4 PM. Thirty minutes before the assessment, participants were instructed to use the restroom and to stand upright for approximately five minutes before testing. Immediately before the examination, participants were requested to remove all metallic accessories and jewelry. During the measurement process, the palms and soles were wiped with a damp cloth to ensure proper current conductivity. Participants were asked to remain silent throughout the procedure.

### 2.4. Muscle Strength

Grip strength was subsequently assessed, which is an essential parameter in the diagnostic process of sarcopenia, serving as an indicator of reduced muscular strength. Measurements were performed using a handheld dynamometer, an instrument designed to measure the force exerted. The operating principle of the dynamometer typically relies on Hooke’s law, which states that the deformation of an elastic element is proportional to the force applied. The unit of measurement for grip strength is conventionally expressed in kilograms (kg), with the scale extending up to 94 kg, and the graduations set at 2 kg intervals. For each participant, grip strength was measured on both sides (dominant and non-dominant hands) to account for bilateral differences. The highest value from three consecutive measurements was recorded for analysis, with a rest period of 30 s between trials to minimize fatigue. This standardized approach is widely employed to evaluate muscle function and strength in clinical assessments and provides reliable data for diagnosing sarcopenia and monitoring overall muscle health.

### 2.5. Statistical Analysis

The data analysis was performed using the Real Statistics Resource Pack software (Release 8.9.1; Copyright (2013–2023) Charles Zaiontz; www.real-statistics.com) and the MetaboAnalyst 6.0 software (https://www.metaboanalyst.ca/). Multiple linear regression was performed with Statistics Kingdom online tool (https://www.statskingdom.com/410multi_linear_regression.html, accessed on 12 July 2024). Normality of distribution was verified with a Shapiro–Wilk test. Variables with normal distributions are presented as mean ± standard deviation (SD) and those without normal distribution were presented as medians with interquartile range (IQR). Comparisons between two groups were performed with an unpaired *t*-test for equal or unequal variance, depending on the results of Fisher test, or for non-normally distributed data with U Mann–Whitney tests. For normally distributed data, the effect size was calculated using Cohen’s d, while for non-normally distributed data, the effect size r was used. For multiple-group comparisons, one-way analysis of variance on ranks (ANOVA) was conducted, followed by all pairwise multiple comparison procedures (Dunn’s Method). To check for the associations between variables, Spearman correlation coefficients were calculated. Post hoc power was calculated using G*Power ver. 3.1.9.7 for the Wilcoxon–Mann–Whitney test for two independent groups with two tails and the A.R.E. method.

## 3. Results

### 3.1. Descriptive Data

The study groups comprised 103 people, of which 53 were diagnosed with HS and 50 did not present with signs and symptoms of this inflammatory dermatosis. In the HS group, 47% of participants were women, while in the control group, 68% were women. The median age of the HS group was 38 years (range: 19–64), while the control group had a median age of 26 years (range: 20–76). However, the mean BMI was significantly higher in the HS group (29.6 ± 11.1 kg/m^2^) compared to the control group (22.1 ± 5.05 kg/m^2^), with a significant difference (*p* < 0.001, effect size −0.581), as presented in Table 1.

The body composition measurement revealed differences between the groups. The patients with HS had significantly higher fat, water and muscle measures. The BIA revealed higher fat percentage, fat mass, fat-free mass and predicted muscle mass on average in the HS group when compared to healthy control subjects (Supplementary Table S1). The measurements of the body composition and basal metabolic rate are presented in Table 1.

**Figure 1:**
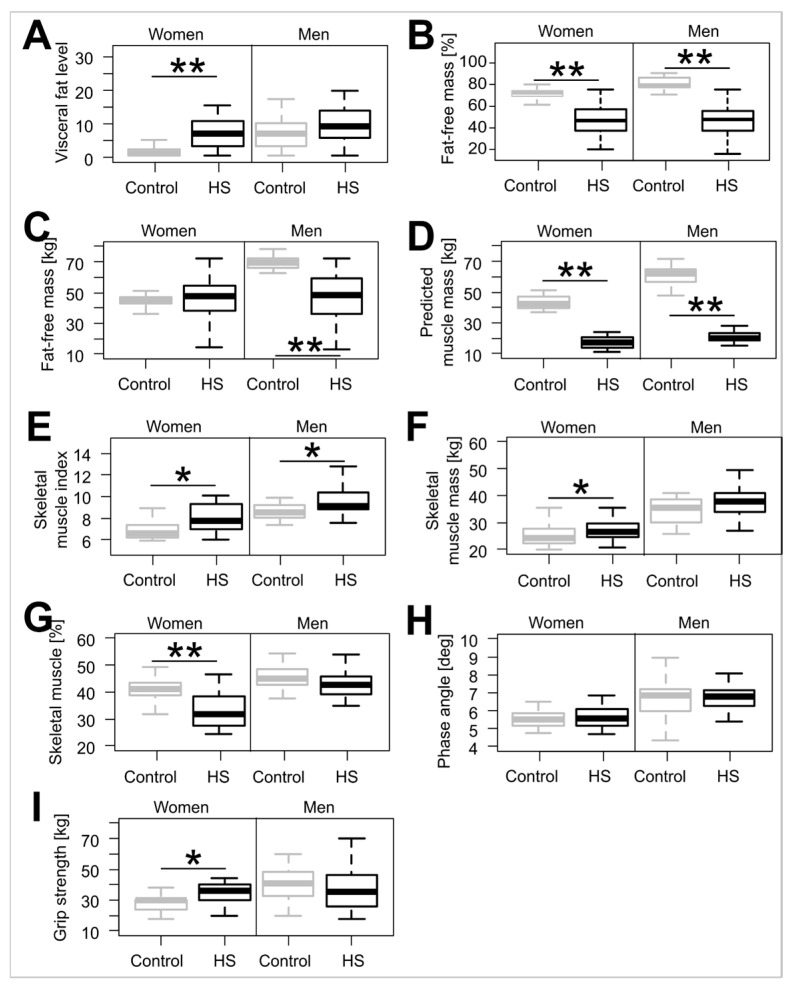
Fat and muscle BIA measures in patients with hidradenitis suppurativa divided by sex (**A**)—visceral fat level; (**B**)—fat-free mass percentage; (**C**)—fat-free mass; (**D**)—predicted muscle mass; (**E**)—skeletal muscle index; (**F**)—skeletal muscle mass; (**G**)—skeletal muscle percentage; (**H**)—phase angle; (**I**)—grip strength. In plots, the bottom and top boxes are the 1st and 3rd quartile, the bolded horizontal bar represents the median, and the asterisk (*) indicates a *p*-value of <0.05. (**) indicates a *p*-value of <0.001 from the U Mann–Whitney test. Abbreviations: HS—hidradenitis suppurativa. Exact *p*-values and effect sizes are given in Supplementary Table S2.

The study group was further divided into men and women on the basis of the well-documented sex disparities in the body composition (Figure 1 and Table 2). The analysis of skeletal muscle measures obtained via BIA showed that while among women, the significant differences in BIA results between HS and the control group persisted, this did not hold true for a majority of measured variables in men. There was a moderate positive relationship between skeletal muscle mass and grip strength in women (Spearman’s rho 0.53, *p* < 0.001) and men (Spearman’s rho 0.53, *p* < 0.001) and between skeletal muscle percentage in women (Spearman’s rho 0.61, *p* < 0.001) and men (Spearman’s rho 0.52, *p* < 0.01). In both men and women, the phase angle (Figure 1G) did not differ significantly between those suffering from HS and those without it (*p*-value for women was 0.844 and for men was 0.845). In women, the fat-free mass and the predicted muscle mass were higher in HS patients, while in men, only the limb-predicted skeletal mass was statistically changed with HS (Table 2).

### 3.2. Quality of Life in HS Patients

Since we noted that the HS seems to affect the BIA results differently depending on sex, we then considered the disease severity and the quality of life in affected patients. Table 3 presents the results of the Hurley grading system for HS severity. Among HS patients, 24% of women and 14% of men presented with stage I disease, 64% of women and 79% of men presented with stage II disease, and 12% of women and 7% of men presented with stage III disease. There were no significant differences (*p* = 0.701) in Hurley score between sexes; however, it should be noted that the effect size of this result was small. Only three patients, all women, scored 4 or higher on the SARF-F evaluation, which indicated that the HS patients generally did not present with symptoms suggestive of sarcopenia. The patients completed two quality of life (QoL) questionnaires, the results of which indicated that most patients experience mild and moderate effects of the HS on their QoL. None of the conducted questionnaires indicated differences in severity and life satisfaction between men and women.

**Figure 2:**
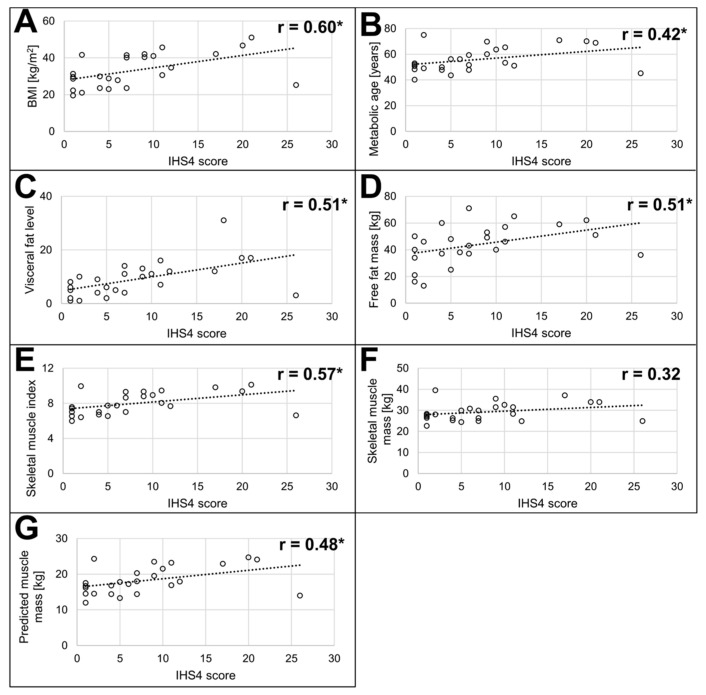
Correlations between BIA measurements and IHS4 score in women. (**A**) body mass index; (**B**) metabolic age; (**C**) visceral fat level; (**D**) fat-free mass; (**E**) skeletal muscle index; (**F**) skeletal muscle mass; (**G**) predicted muscle mass. In bold—Spearman’s correlation coefficient with the asterisk (*) indicating a *p*-value of <0.05.

Next, we attempted to answer the question of whether the BIA results correlate with the patients’ quality of life of HS severity. The strongest statistical relationship was observed between BIA results and IHS4 score for women (Figure 2), where the IHS4 score correlated moderately (Spearman’s rho between 0.5 and 0.7, *p* < 0.05) with women’s BMI, visceral fat level, fat-free mass, and skeletal muscle index and negatively with the percentage of skeletal muscle mass. Some of these associations remained when the whole HS group was considered (Supplementary Table S4), namely between the IHS4 score and BMI, fat-free mass, and skeletal muscle index, but they were weaker (Spearman’s correlation coefficient between 0.31 and 0.33, at *p* < 0.05). We did not find high levels of correlation between QoL questionnaires and men’s BIA results, with the exception of HiSQOL and fat-free mass (Spearman’s rho = 0.38, *p* < 0.05).

The BIA results were not sufficient to distinguish between patients experiencing moderate and mild and severe disease effects on their QoL. In principal component analysis (PCA), the BIA scores did not allow for the grouping of patients with different questionnaire score levels (as described in Table 3), and the one-way analysis of variance (ANOVA) did not identify significant differences between severity groups. We also attempted to build multiple linear regression models from independent BIA data. For HiSQOL, significant predictors were the left leg-predicted muscle mass and skeletal muscle index, explaining the 13.6% variance in the HiSQOL score. The 12.8% variance in DILQ scores was explained by skeletal muscle index and extracellular water. And finally, there was a weak collective non-significant effect between sex, logECW, logBMI, sqrt visceral fat level and IHS4 score, where the model explained 5.3% of the variance. However, it should be noted that the data suffered from homoscedasticity, even when the transformation was conducted, so these models could not be validated.

## 4. Discussion

The HS group exhibited a significantly higher median BMI (29.6 vs. 22.1, *p* < 0.001), reflecting a stronger association of HS with overweight or obesity. The median age of patients with HS was 38 years, but their metabolic age, assessed by comparison of an individual’s BMR with the average BMR of others in the same chronological age group, was notably higher, averaging 65 years. They were characterized by significantly increased levels of body fat mass, visceral fat, basal metabolic rate, and percentage of total body water, along with elevated intracellular and extracellular water, reflecting possible inflammation, fluid retention or metabolic changes. Skeletal muscle mass was higher in the HS group; however, skeletal muscle percentage was significantly lower (38.2% vs. 42.3%, *p* = 0.008). This suggests diminished muscle quality relative to fat in HS patients. The lack of a significant difference in phase angle, a marker of cellular integrity, between the groups suggests that cell health may not differ significantly despite other systemic disparities. Our review of the literature revealed a limited number of studies examining body composition in patients with HS [24,51,52]. The findings of our study show both similarities and discrepancies compared to previously published data; however, the majority of existing studies report increased fat mass and a reduced muscle mass in HS patients [24,51,52]. Variability in results may stem from differences in study populations, methodologies, and settings, particularly among studies utilizing BIA to assess body composition.

The largest study was conducted by Miller et al. [51] on the Danish population. The study included 32 hospital-based patients with HS, 430 population-based patients with HS, and 20,780 controls without HS. Their results showed that patients with HS had a higher fat percentage compared to healthy controls, a higher level of visceral fat, and a lower muscle percentage, similarly to our results. After adjusting for sex and age, Miller et al. [51] found the muscle mass, fat-free mass, and bone mass relative to height (SMI, FFMI, and bone mass/m^2^) to be significantly higher in the HS group compared to the control group. Additionally, patients with HS exhibited a higher basal metabolic rate. In this study, despite it being conducted on a significantly larger patient cohort, the results are similar to those found in our research, particularly concerning the visceral fat and skeletal muscle percentage in patients with HS compared to the healthy population.

Romaní et al. [52] conducted an investigation on body fat composition in individuals with HS treated in a hospital setting. The study cohort comprised 40 Italian patients who met the Dessau criteria for HS, with diagnoses confirmed by a dermatologist. When compared to healthy controls, patients with HS demonstrated a significantly higher percentage of body fat (*p* = 0.02, medium effect size 0.465). Additionally, although not statistically significant, there was a lower basal metabolic rate observed in the HS group. The findings of this study are consistent with our results, demonstrating a notable disparity in the percentage of body fat between individuals with HS and healthy controls.

Mintoff et al. [24] conducted a comprehensive investigation into the relationship between obesity and HS, conditions known to be interconnected through meta-inflammation and both associated with heightened cardiometabolic risk. While the link between these conditions is well established [12,13,14,53], it is also recognized that cardiometabolic pathology is not uniformly present in obesity; a subset of individuals with excess adiposity maintain a metabolically healthy profile. In this cross-sectional study of 632 participants from a population with a high prevalence of obesity and HS, four body composition phenotypes were categorized based on BMI and metabolic status. The study revealed that individuals with HS generally exhibited a worse metabolic profile compared to controls, including higher indices of central adiposity (e.g., VIA—Visceral adiposity index), elevated systolic blood pressure, markers of insulin resistance, and a greater prevalence of metabolic syndrome. Our analysis assessed only for BMI, which was found to be significantly (*p* < 0.001, medium effect size 0.581) higher in HS-affected individuals. Additionally, in Mintoff et al. [24], when categorized by body composition phenotypes, patients with HS consistently displayed more adverse metabolic profiles compared to matched controls, particularly within the normal weight group, even among those deemed metabolically healthy. Inflammation driven by metabolic syndrome may play a role in the development of HS [53]. A study by Mintoff et al. [24] highlights evidence indicating that inflammation associated with HS may independently exacerbate adverse metabolic characteristics, irrespective of adiposity. Despite that, they did not include data assessments of visceral adiposity or body composition analysis, such as a measurement of muscle mass percentage and body fat percentage. BMI was utilized as a measure of obesity, which may have led to misclassification, particularly in individuals with increased muscle mass or a shorter height. In the future, it would be valuable to include BIA measurements in conjunction with metabolic profiles to elucidate the possible association between HS and metabolic syndrome components.

Researchers are also conducting extensive investigations into the influence of diet on body composition and, consequently, its impact on the progression, severity, and therapeutic management of HS [54,55,56,57,58,59,60,61,62]. Verde et al. [63] investigated the effectiveness of a ketogenic diet with a significantly reduced calorie intake in managing HS. Although the authors did not present raw data on body composition parameters, they analyzed the correlations between Sartorius scores and changes in these parameters after 28 days on the diet. BMI decreased significantly after intervention (*p* < 0.001). Weight loss was accompanied by an increase in the percentage of fat-free mass (*p* = 0.004), and an increase in the percentage of the muscle mass (*p* = 0.009). Barrea et al. [64] aimed to examine the association between body composition, adherence to the Mediterranean diet, and the severity of HS in their case-controlled, cross-sectional study conducted in an outpatient setting in Italy. The study did not confirm significant differences in fat mass, contrary to our results, but did find differences in fat-free mass, in line with our results. The study revealed that individuals with HS exhibited significantly impaired body composition, characterized by a reduced phase angle and lower adherence to dietary guidelines, despite their comparable energy intake to that of controls. Barrea at al. [64] note that a reduced phase angle is a predictor of cellular health, but in our study, we did not observe a significant association between HS and this measurement.

The severity of HS can be influenced by hormonal changes. Evidence suggests that sex hormones, such as estrogen and testosterone, play a pivotal role in the pathophysiology of HS, which may also modulate sex-specific differences body composition. The interplay between hormonal fluctuations, disease activity and its clinical characteristics, particularly in lesion distribution (clinical phenotypes), premenstrual exacerbations, postpartum flare-ups, postmenopausal symptom improvement, and the effectiveness of hormone-based therapies has been widely examined and documented in publications [27,41,65,66,67]. The elevated expression of androgen receptors in the lesional skin, especially in sinus tracts, further underscores this relationship and highlights a potential area for further investigation [68]. These hormonal influences may extend to body composition: estrogen is known to promote subcutaneous fat storage in women [69,70], while testosterone enhances muscle mass in men [71,72]. Such mechanisms could explain our findings of higher fat mass and BMI in women with HS and altered muscle distribution in men. Notably, estrogen’s regulation of subcutaneous adipose tissue and testosterone’s anabolic effects on muscle align with the sex-specific body composition patterns observed in our cohort.

Investigating the interplay between sex hormones and pilosebaceous apocrine units in HS, along with elucidating the mechanisms connecting sex hormones to inflammatory pathways, represents a crucial research priority that could pave the way for the development of targeted and effective treatments [44,68]. Sex-specific differences are also evident in the human immune system. Biological females demonstrate enhanced thymic lymphocyte production compared to biological males, leading to a higher proportion of T and B cells, whereas males show a predominance of monocytes and natural killer (NK) cells [73]. In HS, innate and humoral immune responses are hallmark features of HS, and substantial evidence underscores the pivotal role of biological sex in shaping these immune processes, as documented in numerous scientific publications [25,74,75]. For instance, visceral adipose tissue, which was significantly elevated in our HS cohort, secretes pro-inflammatory adipokines (e.g., leptin) that may exacerbate systemic inflammation, particularly in hormonally active individuals [51,76,77]. Leptin, a key adipokine, is directly correlated with fat mass and drives chronic inflammation through interactions with immune cells and pro-inflammatory cytokines such as TNF-α and IL-6 [76,78]. This aligns with our observation of higher metabolic age and basal metabolic rate in HS patients, suggesting a link between altered body composition, hormonal status, and chronic inflammation.

Our study’s stratification of data by sex revealed that HS manifests differently in men and women, particularly concerning body composition. Women with HS exhibited significant changes in fat-free mass and predicted muscle mass when compared to healthy controls, whereas in men, only the limb skeletal muscle mass differed significantly between HS patients and controls. Notably, regional body composition, particularly the distribution of adipose and lean mass across anatomical segments, may further elucidate metabolic disparities in HS. For instance, trunk adiposity and limb muscle mass have distinct implications for systemic inflammation and insulin resistance, both of which are elevated in HS patients [51]. These findings align with the existing body of research indicating that gender plays a critical role in how HS interacts with metabolic and physiological systems [25,27,44,65]. Importantly, while no significant difference in IHS4 scores was observed between sexes in our cohort, we identified sex-specific correlations between body composition and disease severity. In women, IHS4 scores correlated moderately with BMI, visceral fat and muscle indices (Spearman’s rho between 0.5 and 0.7, *p* < 0.05), whereas in men, only weak associations emerged (Spearman’s correlation coefficient between 0.31 and 0.33, at *p* < 0.05). This divergence suggests that hormonal influences, such as estrogen’s role in modulating immune responses [25] and adipose tissue distribution [27], may predominantly affect metabolic parameters linked to HS severity in women, rather than directly altering clinical severity scores like IHS4. To date, no prior research has addressed the relationship between sex-specific body composition and HS pathogenesis. This gap highlights the novelty of our study, as previous investigations focused on BMI rather than nuanced body composition metrics.

It is unequivocal that HS is intrinsically linked to chronic inflammation, a characteristic it shares with conditions such as obesity and sarcopenia [15,79,80]. The accumulation of body fat is often associated with a reduced proportion of skeletal muscle mass with impaired functions, in extreme cases, can manifest as sarcopenic obesity [81,82]. Sarcopenia is a generalized skeletal muscle disease associated with progressive loss of muscle mass and gradual impairment of muscle function directly related to adverse health outcomes such as falls, fractures, frailty syndrome, and increased mortality [83]. The mentioned pathologies such as increased fat mass and decreased muscle mass are factors associated with HS. There is room for future research in the area of phase index as some researchers indicate that phase index changes with disease severity in diabetes which indicates that longitudinal studies including HS patients and making regularly a whole body composition analysis could also monitor the progression of HS.

Our study highlights the complex interplay between body composition, disease severity, and QoL in patients with hidradenitis suppurativa. While the majority of HS patients reported mild to moderate effects on QoL based on validated questionnaires (HiSQOL and DLQI), no significant differences were observed between men and women in terms of life satisfaction or disease impact severity. This finding aligns with previous research suggesting that HS universally impairs QoL, regardless of sex, although individual experiences may vary [84,85,86]. Interestingly, our results showed that body composition parameters, such as BMI, visceral fat level, fat-free mass, and skeletal muscle index, correlated moderately with disease severity (IHS4 score) in women but not in men. This suggests potential sex-specific differences in how HS affects body composition and its relationship to disease burden. Regarding sarcopenia, only three patients (all women) scored 4 or higher on the SARC-F evaluation, indicating that symptoms suggestive of sarcopenia were generally not prominent in our cohort. However, body composition analysis revealed nuanced associations between muscle health and QoL. For instance, skeletal muscle index and extracellular water emerged as significant predictors of DLQI scores, explaining 12.8% of the variance. Similarly, left leg-predicted muscle mass and skeletal muscle index explained 13.6% of the variance in HiSQOL scores. These findings underscore the potential role of muscle health in influencing QoL outcomes in HS patients, even in the absence of overt sarcopenia. Despite these correlations, our data indicate that BIA results alone were insufficient to distinguish between patients experiencing varying levels of QoL impairment or disease severity. Principal component analysis and one-way ANOVA failed to identify significant clustering or differences between severity groups based on BIA-derived parameters. This suggests that while BIA provides valuable insights into body composition, it may not fully capture the multifaceted nature of QoL impairments or disease burden in HS. Overall, our findings emphasize the need for a multidimensional approach to understanding HS-related QoL impairments and their associations with body composition and muscle health. Future research should explore more robust models incorporating clinical, psychological, and lifestyle factors to better predict QoL outcomes and inform personalized management strategies for HS patients.

This study has several limitations that warrant consideration. First, the male-to-female ratio in the control group (2:1) diverged from the HS group (1:1), possibly reflecting regional demographic trends or sampling biases, such as higher male participation in health studies. This imbalance may have confounded sex-specific comparisons, particularly given known hormonal influences on body composition and inflammation. The older mean age of the HS group (38 vs. 26 years in controls) aligns with the chronic nature of HS and frequent delays in diagnosis [87]. Late-onset HS cases, which disproportionately affect males [88], may further explain this disparity. Age-related factors, such as cumulative metabolic dysfunction or lifestyle changes, could independently influence body composition and inflammation, complicating causal interpretations. Future studies should prioritize age- and sex-matched controls to mitigate confounding effects. Second, while bioelectrical impedance analysis offers practical advantages, its limitations include an inability to differentiate visceral from subcutaneous adipose tissue, a critical distinction given the metabolic risks of visceral adiposity in HS. This methodological constraint limits our ability to fully characterize fat distribution patterns and their relationship to systemic inflammation. Future studies should integrate imaging techniques (e.g., Computer Tomography, Dual-Energy X-ray Absorptiometry) to validate BIA-derived visceral fat estimates. Moreover, while participants followed standardized protocols (temporary fasting, bladder voiding), hydration status was not quantitatively assessed (e.g., via urine specific gravity), potentially affecting body composition metrics. Third, the cross-sectional design precludes causal inferences about whether body composition alterations drive HS progression or result from chronic inflammation. For instance, reduced muscle mass could reflect sarcopenia secondary to inflammation or represent a predisposing factor for HS severity. Longitudinal studies tracking body composition changes before and after HS onset are needed. Finally, unmeasured variables such as diet, physical activity, smoking, and comorbidities (e.g., polycystic ovary syndrome, diabetes) may confound the observed associations. In fact, meta-analyses confirm significant associations between HS and smoking, obesity, and type 2 diabetes mellitus [89]. Furthermore, smoking is linked to increased abdominal obesity [90] and may negatively affect body composition [91], while physical activity and adherence to a Mediterranean diet may improve HS severity [61,62]. These omissions limit our understanding of how lifestyle and clinical factors interact with body composition in HS. Future studies should incorporate detailed assessments of these variables to clarify their roles in disease pathophysiology.

This study is strengthened by the inclusion of a relatively large cohort of patients with HS and a detailed body composition analysis. In contrast to prior research, we present results not only for the whole body but also for the extremities and trunk, providing a more granular assessment of body composition. Given the low prevalence of HS, our findings contribute valuable epidemiological data and advance our understanding of the disease’s complex relationship with body composition. Specifically, our comprehensive assessment of regional body composition may reveal novel insights into localized metabolic disturbances in HS.

## 5. Conclusions

HS is a chronic inflammatory condition strongly associated with an elevated BMI, which contributes to metabolic syndrome and increased cardiovascular morbidity. This relationship is mediated by a chronic systemic inflammatory state linked to both obesity and the pathophysiology of HS. Although BMI remains a widely used screening tool for obesity, it does not capture the complexities of body composition. Our study identifies distinct patterns in body composition among HS patients compared to controls, highlighting the potential for enhanced diagnostic and risk assessment approaches. Sex-specific differences in body composition are particularly notable, with women exhibiting higher fat mass and men demonstrating differential muscle distribution. These variations may be influenced by sex hormones, which regulate fat storage and muscle metabolism. Furthermore, sex impacts immune responses and inflammatory processes in HS, contributing to the heterogeneity of disease presentation. Routine bioimpedance measurements offer a non-invasive method to assess body composition and may assist in identifying individuals at elevated risk for metabolic comorbidities. BIA provides quantitative data on parameters such as fat mass, lean body mass, and water distribution, making it a valuable tool for clinical use. Future research should prioritize sex-stratified analyses to elucidate the interplay between hormonal regulation, immune dysregulation, and body composition in HS. Such investigations may facilitate the development of personalized management strategies aimed at improving patient outcomes.

## Figures and Tables

**Table 1:** Bioelectrical impedance analysis results of control subjects and patients with hidradenitis suppurativa.

Variable	Format	Control Group		HS Group		*p*-Value (Effect Size ^✝^)	Power
		Value	Min–Max Value	Value	Min-Max Value		
Sex	No. female/male	34/16	NA	25/28	NA	NA	NA
Age [years]	Median (IQR)	26 (18)	20–76	38 (16)	19–64	0.075 (−0.239)	0.86
Body mass index (BMI) [kg/m^2^]	Median (IQR)	22.1 (5.1)	17.7–38.7	29.6 (11.1)	19.5–51	<0.001 (−0.581)	0.93
Height [cm]	Mean (SD)	171 (8.9)	145–193	173 (8.1)	151–191	0.451 (−0.074)	0.48
Body mass [kg]	Median (IQR)	69.3 (15.6)	48.6–119.8	93.7 (23.5)	51.7–161.6	<0.001 (−0.550)	0.10
Body fat [%]	Mean (SD)	26.0 (7.5)	9.5–41.2	30.8 (18.1)	7.4–49.8	0.020 (0.465)	0.65
Body fat [kg]	Median (IQR)	16.3 (6.0)	5.9–45.2	26.2 (22.7)	5–71.4	<0.001 (−0.400)	0.71
Visceral fat level (TANITA rating scale)	Median (IQR)	2 (6)	1–19	9 (8)	1–31	<0.001 (−0.473)	0.89
Fat-free mass [kg]	Median (IQR)	45.4 (21.3)	35–76.5	46.0 (21.0)	12–71	0.064 (−0.183)	0.15
Fat-free mass [%]	Median (IQR)	73.3 (8.12)	58–90	46.7 (21.0)	12–17	<0.001 (−0.765)	0.73
Metabolic age [years]	Median (IQR)	21 (20)	12–73	65 (18)	40.2–90.2	<0.001 (−0.760)	0.99
Total body water (TBW) [%]	Median (IQR)	31.9 (14.3)	23.9–56.2	45.9 (12.3)	28.9–67.1	<0.001 (−0.508)	0.85
Predicted muscle mass [kg]	Median (IQR)	47.3 (18.8)	37.2–72.8	19.8 (5.8)	12–73.8	<0.001 (−0.740)	0.95
Extracellular Water (ECW) [kg]	Median (IQR)	19.7 (7.0)	12.2–34.9	24.3 (8.8)	16.9–38.3	<0.001 (−0.350)	0.60
Intracellular Water (ICW) [kg]	Median (IQR)	23.1 (10.3)	12.3–35.3	58.1 (18.9)	24.4–85.8	<0.001 (−0.828)	0.99
Skeletal muscle index (SMI) [kg/m^2^]	Median (IQR)	7.3 (2.0)	5.87–9.85	8.9 (2.1)	5.98–14.11	<0.001 (−0.445)	0.76
Skeletal muscle mass [kg]	Median (IQR)	29.0 (10.9)	21.7–42.8	33.9 (11.5)	22.6–51.3	<0.001 (−0.332)	0.55
Skeletal muscle [%]	Mean (SD)	42.3 (5.5)	30.5–57.7	38.2 (7.8)	24.2–56	0.008 (−0.263)	0.25
Bone mass [kg]	Median (IQR)	2.5 (1.0)	2–3.8	3.2 (0.8)	2.1–4.4	<0.001 (−0.421)	0.73
Basal metabolic rate (BMR) [kJ]	Median (IQR)	6232 (1887)	4991–9496	8068 (2432)	5095–12,037	<0.001 (−0.444)	0.76
Basal metabolic rate (BMR) [kcal]	Median (IQR)	1489 (451)	1192–2268	1927 (581)	1217–2875	<0.001 (−0.444)	0.76
Phase angle [deg]	Median (IQR)	5.8 (1.4)	4.4–9	6.2 (1.2)	4.7–8.1	0.219 (0.121)	0.19
Grip strength [kg]	Median (IQR)	30 (10)	18–60	36 (14)	18–70	0.153 (0.141)	0.22

^✝^ effect size is Cohen’s d for normally distributed variables shown as median (SD) and Mann–Whitney r for variables lacking normal distribution shown as median (IQR). IQR, interquartile range; SD, standard deviation; NA, not applicable. Additional parameters were derived based on data obtained from bioelectrical impedance analysis (BIA) and anthropometric measurements: Skeletal muscle index (SMI) (kg/m^2^) = (muscle mass (kg)/height × height (m^2^)). Muscle percentage (%) = (muscle mass (kg)/body weight (kg)) × 100. Body mass index (BMI) (kg/m^2^) = body weight (kg)/height × height (m^2^). Body fat percentage (%) = (fat mass (kg)/body weight kg) × 100. Fat-free mass percentage (%) = (fat-free mass (kg)/body weight (kg)) × 100. TANITAs definition of muscle mass = skeletal muscles + smooth muscles + the water stored in the muscles. TANITAs definition of fat-free mass: muscle, bone, and water. TANITAS definition of bone mass: the quantity of bone tissue in the body, including bone minerals such as calcium and other essential inorganic components.

**Table 2:** Skeletal muscle measures from bioelectrical impedance analysis in limbs and trunks of control subjects and HS patients with hidradenitis suppurativa divided by sex.

Variable ^†^	Women					Men				
	Control Group		HS Group		*p*-Value (Effect Size ^‡^)	Control Group		HS Group		*p*-Value (Effect Size ^‡^)
	Value ^†^	Min–Max	Value ^†^	Min–Max		Value ^†^	Min–Max	Value ^†^	Min–Max	
No. of participants	34	NA	25	NA	NA	28	NA	14	NA	NA
Age [years]	24 (6)	20–55	36 (19)	19–56	0.056 (−0.300)	55 (30)	20–76	40 (19)	20–64	0.160 (−0.160)
BMI [kg/m^2^]	20.6 (3.7)	17.7–28.3	30.6 (16.2)	19.5–51.0	<0.001 (−0.672)	25.3 (4.4)	21.8–38.7	28.7 (6.9)	20.8–50.4	0.067 (−0.276)
Height [cm]	169 (6.5)	145–180	167 (7.0)	151–176	0.409 (−0.119)	178 (4.5)	165–191	182 (7)	173–193	0.152 (−0.216)
Body mass [kg]	60.3 (8.2)	48.6–75.5	87.3 (48.1)	51.7–139.7	<0.001 (−0.798)	92.9 (24.7)	57.9–161	85.1 (13.3)	71.7–120	0.140 (−0.223)
Body fat [%]	27 (6.9)	12.1–41.2	40.5 (14.5)	22.2–49.8	<0.001 (−0.439)	20.4 (8.1)	9.5–38	24.6 (12.7)	7.4–44	0.143 (−0.221)
Metabolic age [years]	21 (5.5)	12–48	52.9 (14.6)	40.2–75	<0.001 (−0.842)	40 (22)	12–73	70 (10)	53–90	<0.001 (−0.674)
Right leg										
Fat-free mass [kg]	7.4 (0.65)	5.9–8.2	8.9 (2.2)	6.7–12.4	<0.001 (−0.631)	11.4 (1.9)	9.7–14.1	12.1 (1.8)	8.4–17.9	0.227 (−0.182)
Predicted muscle mass [kg]	7.4 (1.05)	6–11.1	44.5 (13.2)	28.9–55.8	0.008 (−0.382)	10.3 (2.1)	7.7–12.3	11.5 (1.7)	8–16.9	0.021 (−0.348)
Left leg										
Fat-free mass [kg]	7.4 (0.95)	5.8–70.4	8.6 (2.6)	6.5–12.2	<0.001 (−0.529)	11.2 (1.8)	9.5–13.9	11.9 (2.0)	8.0–18.3	0.204 (−0.191)
Predicted muscle mass [kg]	7.2 (1)	6.1–11.1	8.1 (2.4)	6.1–11.5	0.005 (−0.407)	10.0 (1.5)	7.9–12.3	11.3 (1.9)	7.6–17.3	0.012 (−0.377)
Right arm										
Fat-free mass [kg]	2 (0.25)	1.6–2.5	2.5 (0.7)	1.9–3.5	<0.001 (−0.674)	3.8 (0.7)	3.1–4.7	4.2 (0.9)	2.9–6	0.063 (−0.280)
Predicted muscle mass [kg]	1.9 (0.45)	1.7–3.9	2.4 (0.6)	1.8–3.3	0.001(−0.469)	3.4 (0.9)	2.2–4.2	4.0 (0.8)	2.7–5.6	0.009 (−0.391)
Left arm										
Fat-free mass [kg]	1.9 (0.3)	1.5–2.5	2.6 (1.0)	1.8–3.9	<0.001 (−0.747)	3.9 (0.8)	3.1–4.9	4.2 (0.8)	3–5.7	0.096 (−0.025)
Predicted muscle mass [kg]	25.1 (7.6)	8–41.9	2.5 (0.9)	1.7–3.7	<0.001 (−0.857)	21.7 (10.3)	7.1–41.9	4.0 (0.7)	2.8–5.4	<0.001 (−0.825)
Trunk										
Fat-free mass [kg]	25.5 (2.6)	19.8–31.6	30 (8.1)	23.3–43.1	<0.001 (−0.684)	37.9 (1.8)	35.8–40.3	37.3 (4.1)	30.5–44.4	0.600 (−0.079)
Predicted muscle mass [kg]	24.6 (4.1)	22.1–39.6	28.5 (8)	22.2–41.1	<0.001 (−0.542)	35.7 (3.9)	26.9–38.8	35.7 (3.99)	29.0–42.5	0.311 (−0.153)

^†^ Values are medians with interquartile range in parentheses; ^‡^ Mann–Whitney r; NA, not applicable. Additional variables are included in Supplementary Table S3.

**Table 3:** Results of disease severity classification and quality of life questionnaires among hidradenitis suppurativa patients.

Staging/Questionnaire Score ^†^	Whole Study Group		Women		Men		Men vs. Women	
	Value	Min–Max	Value	Min–Max	Value	Min–Max	U Mann–Whitney *p*-Value (Effect Size r)	Chi-Square *p*-Value (Cramer’s V)
Hurley stage ^†^	2(0)	1–3	2 (0)	1–3	2 (0)	1–3	0.701 (−0.053)	0.501 (0.162)
Stage I [no]	10		6		4		NA	NA
Stage II [no]	38		16		22		NA	NA
Stage III [no]	5		3		2		NA	NA
IHS4 score ^†^	6 (7)	1–55	7 (9)	1–26	6 (7)	1–55	0.900 (−0.017)	0.866 (0.072)
Mild [no]	13		7		6		NA	NA
Moderate [no]	28		13		15		NA	NA
Severe [no]	12		8		7		NA	NA
SARC-F score ^†^	1 (2)	0–4	1 (3)	0–4	1 (1)	0–3	0.101 (−0.226)	0.246 (0.159)
Score < 4 [no]	50		22		28		NA	NA
Score ≥ 4 [no]	3		3		0		NA	NA
DLQI score ^†^	4 (7)	0–17	5 (8)	0–17	4 (5)	1–12	0.674 (−0.058)	0.253 (0.277)
No effect [no]	15		8		7		NA	NA
Small effect [no]	17		5		12		NA	NA
Moderate effect [no]	14		7		7		NA	NA
Very large effect [no]	7		5		2		NA	NA
Extremely large effect [no]	0		0		0		NA	NA
HiSQOL score ^†^	13 (12)	0–31	13 (12)	0–31	11 (11)	1–25	0.858 (−0.025)	0.823 (0.170)
None [no]	15		8		7		NA	NA
Mild [no]	17		6		11		NA	NA
Moderate [no]	15		8		7		NA	NA
Severe [no]	1		0		1		NA	NA
Very severe [no]	5		3		2		NA	NA

^†^ Values are medians with interquartile range in parentheses; no, number of patients; NA, not applicable.

## Data Availability

The data that support the findings of this study are available on request from the corresponding author.

## Supplementary Materials

The following supporting information can be downloaded at https://www.mdpi.com/article/10.3390/jcm14082760/s1, Table S1: Bioelectrical impedance analysis results of control subjects and patients with hidradenitis suppurativa—fat and muscle measures in limbs and trunk; Table S2: Exact *p*-values for comparisons between fat and muscle BIA measures in patients with hidradenitis suppurativa divided by sex; Table S3: Additional measures from bioelectrical impedance analysis in limbs and trunk of control subjects and hidradenitis suppurativa patients divided by sex. Table S4: Spearman’s correlation coefficients for bioelectrical impedance analysis and severity classification and quality of life questionnaires among patients with hidradenitis suppurativa.

## Abbreviations

The following abbreviations are used in this manuscript:

ANOVA	One-way analysis of variance on ranks
BIA	Bioelectrical impedance analysis
BMI	Body mass index
BMR	Basal metabolic rate
DLQI	Dermatology Life Quality Index
ECW	Extracellular water
FFMI	Fat-free body mass index
HiSQOL	Hidradenitis Suppurativa Quality of Life
HS	Hidradenitis suppurativa
IHS4	International Hidradenitis Suppurativa Severity Score System
IL-6	Interleukin 6
IQR	Interquartile range
NK cells	Natural killer cells
SARC-F	Strength, assistance with walking, rising from a chair, climbing stairs, and fall
SD	Standard deviation
SMI	Skeletal muscle index
TNF-α	Tumor necrosis factor-alpha
VIA	Visceral adiposity index
