# Supplementary material for: The Role of Sex in Body Composition Differences in Hidradenitis Suppurativa: Insights from Bioelectrical Impedance Analysis
Source: J Clin Med. 2025 Apr 17;14(8):2760. doi: 10.3390/jcm14082760 (PMC12028129; doi:10.3390/jcm14082760)
Supplement: Supplementary file 1 [file jcm-14-02760-s001.zip › jcm-3480170-supplementary.pdf]

Supplementary Materials

Table S1. Bioelectrical impedance analysis results of control subjects and patients with hidradenitis suppurativa—fat and muscle measures in limbs and trunks.

Abbreviated Variable	Variable	Format	Control group		HS group	
			Value	Min-max value	Value	Min-max value
	**Right leg**					
RL FatP	**Fat percentage [%]**	Mean (SD)	29.8(13.575)	6.8 - 40.8	31.2(13.6)	3.9 - 55.8
RL FatM [kg]	**Fat mass [kg]**	Median (IQR)	3.1(1.15)	0.9 - 7.2	4(3.9)	0.5 - 13.8
RL FFM [kg]	**Fat-free mass [kg]**	Median (IQR)	7.65(3.15)	5.9 - 14.1	10.8(3.4)	6.7 - 17.9
RL PMM [kg]	**Predicted muscle mass [kg]**	Median (IQR)	7.8(2.9)	6 - 12.3	10.2(3.2)	6.3 - 16.9
	**Left leg**					
LL FatP [%]	**Fat percentage [%]**	Mean (SD)	27.5(8.5)	7.2 - 40.4	31.5(13.3)	5.2 - 55.4
LL FatM [kg]	**Fat mass [kg]**	Median (IQR)	3.1(1.2)	0.9 - 6.3	4(3.7)	0.6 - 13.5
LL FFM [kg]	**Fat-free mass [kg]**	Median (IQR)	7.6(3.4)	5.7 - 70.4	10.9(3.4)	6.5 - 18.3
LLPMM [kg]	**Predicted muscle mass [kg]**	Median (IQR)	7.7(2.8)	6.1 - 12.3	10.3(3.3)	6.1 - 17.3
	**Right arm**					
RA FatP [%]	**Fat percentage [%]**	Median (IQR)	24.9(7.8)	12.4 - 44.6	28.5(21.9)	9.7 - 63.3
RA FatM [kg]	**Fat mass [kg]**	Median (IQR)	0.7(0.4)	0.4 - 3.2	1.3(1.5)	0.4 - 5.7
RA FFM [kg]	**Fat-free mass [kg]**	Median (IQR)	2.1(1.4)	1.6 - 4.7	3.4(1.8)	1.9 - 6
RAPMM [kg]	**Predicted muscle mass [kg]**	Median (IQR)	2.2(1.3)	1.6 - 4.5	3.2(1.6)	1.8 - 5.6
	**Left arm**					
LA FatP [%]	**Fat percentage [%]**	Median (IQR)	25.8(7.8)	12.2 - 42.9	29.5(23.7)	9.8 - 64.2
LA FatM [kg]	**Fat mass [kg]**	Median (IQR)	0.7(0.4)	0.4 - 3.2	1.5(1.9)	0.3 - 6.6
LA FFM [kg]	**Fat-free mass [kg]**	Median (IQR)	2(1.4)	1.4 - 4.9	3.7(1.5)	1.8 - 5.7
LA PMM [kg]	**Predicted muscle mass [kg]**	Median (IQR)	25.1(8)	7.1 - 41.9	3.5(1.4)	1.7 - 5.4
	**Trunk**					
TR FatP [%]	**Fat percentage [%]**	Mean (SD)	25.1(8.0)	7.1 - 41.9	29.3(10.2)	5.9 - 50.1
TR FatM [kg]	**Fat mass [kg]**	Median (IQR)	88(5.1)	2.1 - 27.7	14.9(10.2)	1.9 - 42.4
TR FFM [kg]	**Fat-free mass [kg]**	Median (IQR)	26.3(11.8)	19.8 - 40.3	35.6(8.8)	23.3 - 44.4
TR PMM [kg]	**Predicted muscle mass [kg]**	Median (IQR)	26.7(10.7)	21.4 - 40.2	34(8.6)	22.2 - 42.5

Table S2. Exact p-values for comparisons between fat and muscle BIA measures in patients with hidradenitis suppurativa divided by sex.

	**Women with HS vs. control p-value (effect size) †**		**Men with HS vs. control p-value (effect size) †**
	Raw	Body mass-adjusted‡	Raw
**Visceral fat level**	< 0.001 (-0.593)	< 0.001 (-0.501)	0.163 (-0.210)
**Fat-free mass [%]**	< 0.001 (-0.759)	< 0.001 (-0.825)	< 0.001 (-0.780)
**Fat-free mass [kg]**	0.597 (-0.069)	< 0.001 (-0.759)	< 0.001 (-0.666)
**Predicted muscle mass [kg]**	< 0.001 (-0.856)	< 0.001 (-0.849)	< 0.001 (-0.666)
**Skeletal muscle index**	0.003 (-0.423)	< 0.001 (-0.575)	0.010 (-0.388)
**Skeletal muscle mass [kg]**	0.011 (-0.366)	< 0.001 (-0.637)	0.110 (-0.241)
**Skeletal muscle [%]**	< 0.001 (-0.539)	< 0.001 (-0.599)	0.090 (-0.265)
**Phase angle [deg]**	0.844 (-0.028)	< 0.001 (-0.603)	0.845 (-0.029)
**Grip strength [kg]**	0.019 (-0.340)	0.007 (-0.353)	0.187 (-0.199)

† p-values from U Mann–Whitney test; effect size of Cohen's d; the effect size is Mann–Whitney r, ‡ adjustment was carried out by performing a U Mann–Whitney test on residuals extracted from the regression of variables in a row ~ body mass

Table S3. Additional measures from bioelectrical impedance analysis in limbs and trunks of control subjects and hidradenitis suppurativa patients divided by sex.

Variable†	Women					Men			
	Control group		HS group		p - value (effect size‡)	Control group		HS group	
	Value †	Min-max value	Value †	Min-max value		Value †	Min-max value	Value †	Min-max value
Total body water (TBW) [%]	30.7(2.75)	24.8 - 36.1	38(10.4)	28.9 - 53.8	<0.001 (-0.755)	48.1(5.0)	41.5 - 56.2	49.9(7.4)	37.8 - 67.1
Extracellular Water (ECW) [kg]	18.8(8.3)	12.2 - 34.9	21.1(3.9)	16.9 - 29.5	0.759 (-0.040)	19.5(1.6)	16.3 - 23.6	28.85(7.0)	19.8 - 38.3
Intracellular Water (ICW) [kg]	19.4(4.4)	12.3 - 35.3	50.2(13.9)	38.1 - 71.2	< 0.001 (-0.849)	28.5(3.2)	23.2 - 35.1	65.5(15.3)	24.4 - 85.8
Bone mass [kg]	2.3(0.35)	2.0 - 3.6	2.7(0.7)	2.1 - 3.8	< 0.001 (-0.483)	3.3(0.4)	2.6 - 3.7	3.5(0.5)	2.7 - 4.4
Basal metabolic rate (BMR) [kJ]	5715(910)	4991 - 9106	6703(2144)	5095 - 9781	< 0.001 (-0.482)	7984(936)	6226 - 9433	8572.5(1271)	6230 - 12037
Basal metabolic rate (BMR) [kcal]	1365(218)	1192 - 2175	1601(512)	1217 - 2336	< 0.001 (-0.482)	1907(224)	1487 - 2253	2048(304)	1488 - 2875
Right leg									
Fat percentage [%]	31.1(7)	17.3 - 40.8	44.5(13.2)	28.9 - 55.8	< 0.001 (-0.690)	16.7(5.2)	6.8 - 35.6	19.5(9.3)	3.9 - 35.3
Fat mass [kg]	3.2(1.2)	1.6 - 4.6	6.7(5.4)	2.9 - 13.8	< 0.001 (-0.681)	2.2(0.93)	0.9 - 7.2	3.1(1.8)	0.5 - 9.8
Left leg									
Fat percentage [%]	32(6.6)	3.2(0.95)	44.6(12.6)	29.3 - 55.4	< 0.001 (-0.626)	16.75(6.3)	7.2 - 32.1	20.8(10.0)	5.2 - 33.3
Fat mass [kg]	15.9 - 40.4	1.4 - 4.7	6.7(5.3)	2.8 - 13.5	< 0.001 (-0.684)	2.3(1.0)	0.9 - 6.3	3.2(2.0)	0.6 - 9.1
Right arm									
Fat percentage [%]	25.9(7.0)	0.7(0.4)	46(19.7)	22.2 - 63.3	< 0.001 (-0.623)	18.7(5.2)	12.4 - 44.6	21.7(9.6)	9.7 - 43.3
Fat mass [kg]	18.3 - 38.9	0.4 - 1.1	2(2.5)	0.5 - 5.7	< 0.001 (-0.683)	0.85(0.3)	0.6 - 3.2	1.3(0.7)	0.4 - 4.6
Left arm									
Fat percentage [%]	27.6(6.9)	16.9 - 40.2	46.1(19.3)	22.9 - 64.2	< 0.001 (-0.558)	20.6(5.9)	12.2 - 42.9	22.6(9.8)	9.8 - 49.3
Fat mass [kg]	0.7(0.4)	0.4 - 1.2	2.1(3)	0.5 - 6.6	< 0.001 (-0.670)	0.95(0.4)	0.6 - 3.2	1.3(0.63)	0.3 - 5.5
Trunk									
Fat percentage [%]	25.1(7.6)	8 - 41.9	31.9(14.8)	14.5 - 45.8	0.011 (-0.330)	21.7(10.3)	7.1 - 41.9	27.5(11.9)	5.9 - 50.1
Fat mass [kg]	8.7(4.9)	2.1 - 15.1	17.4(13.2)	4.8 - 31.4	< 0.001 (-0.434)	10.5(6.13)	2.8 - 27.7	14.35(7.3)	1.9 - 42.4

† Values are medians with interquartile range in parentheses,‡ Mann–Whitney r.

Table S4. Spearman's correlation coefficients for bioelectrical impedance analysis and severity classification and quality of life questionnaires among patients with hidradenitis suppurativa.

		**Whole HS group**					**Women**					**Men**		
		**Hurley**	**SARC-F**	**IHS4**	**DLQI**	**HiSQOL**	**Hurley**	**SARC-F**	**IHS4**	**DLQI**	**HiSQOL**	**Hurley**	**SARC-F**	**IHS4**

Age [years]	**Age [years]**	0.18	0.15	0.03	0.16	**0.27***	0.38	0.18	0.37	0.20	0.32	-0.07	0.22	-0.25
BMI [kg/m2]	**BMI [kg/m^2]**	0.17	0.12	**0.36***	0.08	0.10	0.34	0.18	**0.60***	0.03	-0.01	0.02	0.13	0.18
V FatL	**Visceral fat level**	0.21	0.10	**0.31***	0.13	0.18	**0.41***	0.17	**0.60***	0.02	0.01	0.00	0.19	0.07
FFM [kg]	**Fat-free mass [kg]**	0.24	0.15	0.21	0.20	0.26	**0.44***	0.07	**0.51***	0.13	0.13	0.02	0.29	-0.03
MetaAge [years]	**Metabolic age [years]**	0.05	-0.14	0.26	-0.06	-0.06	0.13	0.18	**0.42***	-0.14	-0.18	-0.05	-0.28	0.21
PMM [kg]	**Predicted muscle mass [kg]**	0.13	0.05	**0.31***	0.01	0.07	0.20	0.13	**0.48***	-0.13	-0.15	0.04	0.06	0.16
SMI	**Skeletal muscle index**	0.15	-0.05	**0.33***	0.07	0.13	0.32	0.16	**0.57***	-0.01	-0.01	0.01	0.01	0.19
SSM [kg]	**Skeletal muscle mass [kg]**	0.03	-0.19	0.20	-0.09	-0.07	0.01	0.25	0.32	-0.12	-0.19	0.01	-0.31	0.27
SSM%	**Skeletal muscle [%]**	-0.18	-0.24	-0.24	-0.15	-0.13	**-0.40***	-0.09	**-0.51***	-0.01	0.03	-0.06	-0.27	-0.12
Phase [deg]	**Phase angle [deg]**	0.04	-0.13	0.00	0.04	0.02	0.21	0.23	0.12	0.38	0.28	-0.18	-0.23	-0.22
DYNAM	**Grip strength [kg]**	**0.37***	-0.03	**0.32***	0.03	0.01	0.33	0.26	0.27	-0.09	-0.13	**0.40***	-0.30	0.34
	Right leg:													
RL FFM	**Fat-free mass [kg]**	0.07	-0.17	0.26	-0.06	-0.05	0.14	0.16	**0.42***	-0.19	-0.23	0.06	-0.14	0.35
RL PMM	**Predicted muscle mass [kg]**	0.07	-0.17	0.25	-0.06	-0.05	0.14	0.16	**0.42***	-0.19	-0.23	0.06	-0.14	0.35
	Left leg:													
LL FFM	**Fat-free mass [kg]**	0.05	-0.15	0.27	-0.05	-0.05	0.12	0.16	**0.42***	-0.17	-0.22	0.01	-0.17	0.32
LLPMM	**Predicted muscle mass [kg]**	0.05	-0.16	0.26	-0.05	-0.05	0.12	0.16	**0.42***	-0.17	-0.22	0.01	-0.18	0.31
	Right arm:													
RA FFM	**Fat-free mass [kg]**	0.08	-0.18	0.21	-0.05	-0.02	0.20	0.26	**0.47***	-0.01	-0.04	-0.05	-0.29	0.24
RAPMM	**Predicted muscle mass [kg]**	0.08	-0.19	0.21	-0.05	-0.03	0.20	0.24	**0.47***	-0.02	-0.05	-0.05	-0.29	0.24
	Left arm:													
LA FFM	**Fat-free mass [kg]**	0.06	-0.20	0.21	-0.06	-0.03	0.19	0.17	**0.47***	-0.07	-0.09	-0.02	-0.30	0.24
LA PMM	**Predicted muscle mass [kg]**	0.06	-0.20	0.20	-0.07	-0.04	0.19	0.17	**0.47***	-0.07	-0.09	-0.05	-0.31	0.20
	Trunk:													
TR FFM	**Fat-free mass [kg]**	0.06	-0.03	0.26	-0.03	-0.04	0.10	0.23	0.39	-0.07	-0.12	-0.08	-0.30	0.08
TR PMM	**Predicted muscle mass [kg]**	0.06	-0.03	0.26	-0.04	-0.04	0.10	0.22	0.39	-0.07	-0.12	-0.08	-0.30	0.08
